# Supplementary material for: Modeling glioblastoma heterogeneity as a dynamic network of cell states
Source: Mol Syst Biol. 2021 Sep 16;17(9):e10105. doi: 10.15252/msb.202010105 (PMC8444284; doi:10.15252/msb.202010105)
Supplement: Supplementary file 5 — Source Data for Figure 3 [file MSB-17-e10105-s001.zip › Figure3A_sourcedata/GSEA_3065/hallmarks_state3.GseaPreranked.1623416274744/HALLMARK_WNT_BETA_CATENIN_SIGNALING.html]

Details for gene set HALLMARK\_WNT\_BETA\_CATENIN\_SIGNALING[GSEA]

|  || Dataset | state3 |
| Phenotype | NoPhenotypeAvailable |
| Upregulated in class | na\_pos |
| GeneSet | HALLMARK\_WNT\_BETA\_CATENIN\_SIGNALING |
| Enrichment Score (ES) | 0.43687633 |
| Normalized Enrichment Score (NES) | 1.3059486 |
| Nominal p-value | 0.1039261 |
| FDR q-value | 0.13345411 |
| FWER p-Value | 0.691 |
Table: GSEA Results Summary

  

Fig 1: Enrichment plot: HALLMARK\_WNT\_BETA\_CATENIN\_SIGNALING      
 Profile of the Running ES Score & Positions of GeneSet Members on the Rank Ordered List

  

| PROBE | GENE SYMBOL | GENE\_TITLE | RANK IN GENE LIST | RANK METRIC SCORE | RUNNING ES | CORE ENRICHMENT || 1 | NOTCH1 |  |  | 272 | 0.253 | 0.1247 | Yes |
| 2 | CTNNB1 |  |  | 373 | 0.224 | 0.2513 | Yes |
| 3 | PTCH1 |  |  | 743 | 0.165 | 0.3109 | Yes |
| 4 | JAG1 |  |  | 1011 | 0.138 | 0.3652 | Yes |
| 5 | CCND2 |  |  | 1286 | 0.116 | 0.4053 | Yes |
| 6 | NCOR2 |  |  | 1781 | 0.087 | 0.4026 | Yes |
| 7 | ADAM17 |  |  | 1914 | 0.080 | 0.4369 | Yes |
| 8 | MYC |  |  | 2324 | 0.064 | 0.4292 | No |
| 9 | RBPJ |  |  | 2897 | 0.044 | 0.3909 | No |
| 10 | PSEN2 |  |  | 3299 | 0.033 | 0.3655 | No |
| 11 | TCF7 |  |  | 3320 | 0.033 | 0.3833 | No |
| 12 | NUMB |  |  | 3488 | 0.028 | 0.3817 | No |
| 13 | SKP2 |  |  | 3725 | 0.022 | 0.3682 | No |
| 14 | DVL2 |  |  | 4035 | 0.014 | 0.3416 | No |
| 15 | CUL1 |  |  | 4057 | 0.014 | 0.3476 | No |
| 16 | KAT2A |  |  | 4306 | 0.007 | 0.3233 | No |
| 17 | CSNK1E |  |  | 4623 | -0.002 | 0.2883 | No |
| 18 | NCSTN |  |  | 4795 | -0.006 | 0.2723 | No |
| 19 | AXIN1 |  |  | 4838 | -0.007 | 0.2719 | No |
| 20 | LEF1 |  |  | 4928 | -0.009 | 0.2673 | No |
| 21 | MAML1 |  |  | 4989 | -0.011 | 0.2670 | No |
| 22 | GNAI1 |  |  | 5194 | -0.016 | 0.2532 | No |
| 23 | HDAC5 |  |  | 5513 | -0.025 | 0.2319 | No |
| 24 | TP53 |  |  | 5700 | -0.030 | 0.2291 | No |
| 25 | DKK1 |  |  | 6057 | -0.041 | 0.2138 | No |
| 26 | HDAC2 |  |  | 6905 | -0.076 | 0.1637 | No |
| 27 | HEY1 |  |  | 6933 | -0.077 | 0.2084 | No |
Table: GSEA details [plain text format]

  

Fig 2: HALLMARK\_WNT\_BETA\_CATENIN\_SIGNALING: Random ES distribution      
 Gene set null distribution of ES for **HALLMARK\_WNT\_BETA\_CATENIN\_SIGNALING**
